# Prediction of ISUP grading of clear cell renal cell carcinoma using support vector machine model based on CT images

**DOI:** 10.1097/MD.0000000000015022

**Published:** 2019-04-05

**Authors:** Xiaoqing Sun, Lin Liu, Kai Xu, Wenhui Li, Ziqi Huo, Heng Liu, Tongxu Shen, Feng Pan, Yuqing Jiang, Mengchao Zhang

**Affiliations:** a Department of Radiology, China-Japan Union Hospital of Jilin University; b College of Computer Science and Technology, Jilin University; c Department of Orthopaedics, China-Japan Union Hospital of Jilin University, Changchun, Jilin, China.

**Keywords:** clear cell renal cell carcinoma, computed tomography, image feature, radiomics, tomography

## Abstract

**Background:**

To explore whether radiomics combined with computed tomography (CT) images can be used to establish a model for differentiating high grade (International Society of Urological Pathology [ISUP] grade III–IV) from low-grade (ISUP I–II) clear cell renal cell carcinoma (ccRCC).

**Methods:**

For this retrospective study, 3-phase contrast-enhanced CT images were collected from 227 patients with pathologically confirmed ISUP-grade ccRCC (155 cases in the low-grade group and 72 cases in the high-grade group). First, we delineated the largest dimension of the tumor in the corticomedullary and nephrographic CT images to obtain the region of interest. Second, variance selection, single variable selection, and the least absolute shrinkage and selection operator were used to select features in the corticomedullary phase, nephrographic phase, and 2-phase union samples, respectively. Finally, a model was constructed using the optimal features, and the receiver operating characteristic curve and area under the curve (AUC) were used to evaluate the predictive performance of the features in the training and validation queues. A *Z* test was employed to compare the differences in AUC values.

**Results:**

The support vector machine (SVM) model constructed using the screening features for the 2-stage joint samples can effectively distinguish between high- and low-grade ccRCC, and obtained the highest prediction accuracy. Its AUC values in the training queue and the validation queue were 0.88 and 0.91, respectively. The results of the *Z* test showed that the differences between the 3 groups were not statistically significant.

**Conclusion:**

The SVM model constructed by CT-based radiomic features can effectively identify the ISUP grades of ccRCC.

## 1 Introduction

Renal cell carcinoma (RCC) is the most common primary malignancy of the kidney, accounting for about 85% to 90% of renal malignancies. Clear cell renal cell carcinoma (ccRCC) is the most common subtype of RCC, accounting for about 70% of cases.^[1]^ Because of the heterogeneity of ccRCC tumors, different patients with the same type of tumor are likely to have a different prognosis. Among the main determinants of the prognosis, the nuclear grading of the tumor is widely recognized as an important independent factor.^[2–4]^ Therefore, it is particularly important to explore a method that can accurately distinguish the nuclear grading of tumors. Previous studies have shown that models constructed by screening imaging features from medical images can accurately distinguish between the high and low grades of ccRCC, with accuracy ranging from 0.73 to 0.926.^[5–8]^ However, most of these studies used the Fuhrman nuclear grading standard, which was proposed in 1982, as a reference.^[9]^ Recent studies have revealed loopholes in this grading system, resulting in poor reproducibility of tumor ratings,^[4,10,11]^ and no significant difference exists in long-term survival rates between patients rated as grade II and grade III in this standard.^[12]^ This means that it is futile to use image features to distinguish different nuclear grades. The International Society of Urological Pathology (ISUP) standard proposed by the 2012 ISUP conference working group addresses the above issues, and was recommended by the WHO in 2016. This grading method can accurately distinguish grades, and patients at different levels have a different prognosis.^[2,3]^ With this update of the pathologic grading method, it is worth exploring whether the imaging method can accurately predict the grading of ccRCC.

Radiomics is a technique for automatically extracting quantitative features from medical images. It is able to extract hundreds of features from each image, resulting in more detailed quantitative information about tumors than can be obtained from clinically used imaging metrics.^[13]^ In recent years, studies on lung cancer,^[14,15]^ colorectal cancer,^[16]^ renal cell carcinoma,^[6]^ and bladder cancer^[17]^ have shown that radiomics can provide useful information for tumor grading, staging, and prognosis. Based on this, we investigated whether a support vector machine (SVM) model constructed by extracting features from CT images could identify the ISUP grade of ccRCC.

In summary, the purpose of this study is to report whether a CT-based radiomics model can be used to distinguish the grade of ISUP in ccRCC.

## 2 Materials and methods

### 2.1 Patients

The retrospective study was approved by the Ethics Review Committee of our hospital. The requirement for informed consent was waived, because CT image acquisition is part of a routine examination as a noninvasive technique for suspected patients with RCC.

**Figure 1:**
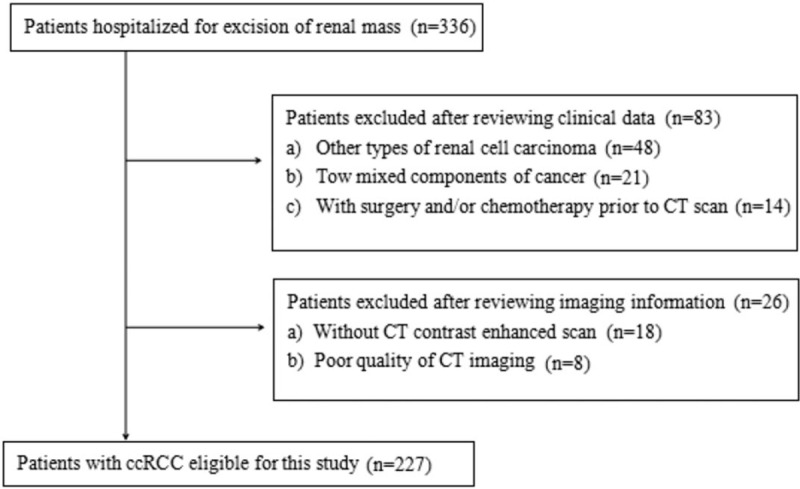
Flow chart of patient recruitment with inclusion and exclusion details.

From March 2014 to March 2018, data were collected from 227 patients (162 men and 65 women, age range 34–77 years) with ccRCC who underwent surgical resection and exhibited pathologic results. Patient inclusion/exclusion details are presented in Figure 1. There was 1 renal mass per patient, so a total of 227 tumors were included in the experiment.

### 2.2 CT examination

Triple-phase CT-enhanced images were obtained using a 64-slice CT scanner (GE Discovery 750 HD BASE (M)). The scanning parameters were as follows: tube voltage of 120 kV, tube current automatic adjustment technology, scanning range of 500.00 mm, scanning thickness of 1.25 mm, rotation speed of 0.6 s/circle, and a 512 × 512 matrix. The patients were injected with a contrast medium (Omnipaque, 300 mg iodine/mL; GE Healthcare, Shenyang, China) via an elbow vein using a high-pressure syringe (Missouri XD 2001; Ulrich Medical, Ulm, Germany) at a rate of 4.5 mL/s for a total of 100 mL. The corticomedullary phase scan was performed 25 to 30 seconds after the injection of the contrast medium, the nephrographic scan was performed at 60 to 70 seconds, and the excretory phase was scanned at 120 to 180 seconds. All patients were scanned while holding their breath after deep inhalation.

### 2.3 Data preprocessing

For the 227 patients, all images were enhanced CT scan images. The image quality and image noise vary depending on the area of the renal cortex. Therefore, it was necessary to preprocess the data to ensure that the image features were calculated with the same specifications. All images were resampled into voxel sizes of 1 × 1 × 1 mm^3^ using linear interpolation. Furthermore, a Gaussian filter was used for denoising. Tumor segmentation and feature extraction were then performed.

### 2.4 Image analysis and feature extraction

In this study, we selected the largest cross-section of the tumor for region of interest (ROI) delineation. The delineation of the ROI was performed by 2 radiologists with 8 and 6 years of CT interpretation experience, respectively, at Radcloud (Huiying Medical Technology Co, Ltd, Beijing, China). During the description, they were unaware of the pathology of the tumor and the results of the imaging results.

**Figure 2:**
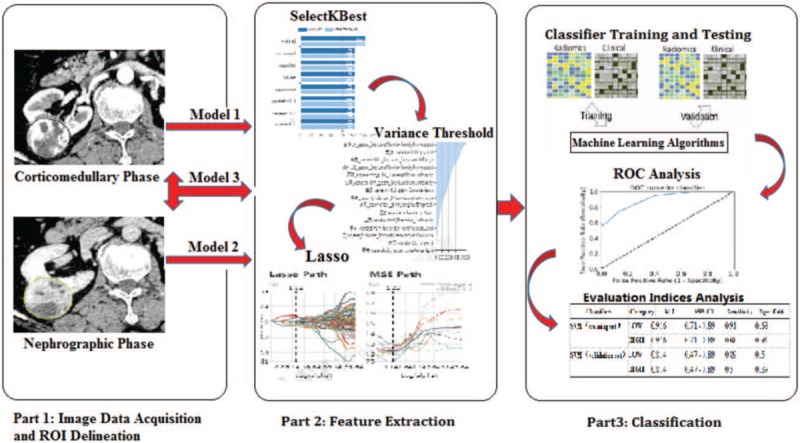
Schematic diagram of feature extraction and radiomics analysis for clear cell renal cell carcinoma grading. ROC = receiver operating characteristic, ROI = region of interest.

For each ROI, we used Radcloud to extract and calculate features. A total of 1029 quantitative imaging features were extracted from the ROI using the Radcloud platform. These features were then placed into 1 of 4 groups. The 1st group consists of 95 first-order statistics that quantify the distribution of voxel intensities within a CT image by common and basic measures. The 2nd group of features is based on shape and size, and contains 15 three-dimensional features reflecting the shape and size of the region. The 3rd group consists of 295 texture features calculated by gray level run length and gray level co-occurrence texture matrix, which can quantify regional heterogeneity differences. The 4th group (higher-order statistical features) includes intensity and texture features obtained from the wavelet transform of the original CT image. Five types of filters were used for feature extraction: exponential, square, square root, logarithmic, and wavelet (wavelet-LLL, wavelet-HHH, wavelet-HLL, wavelet-HHL, wavelet-LLH, wavelet-HLH, wavelet-LHL, wavelet-LHH). To reduce the dimensionality of the features, we used a variance threshold (variance threshold = 0.8), select K best (*P* < .05), and the least absolute shrinkage and selection operator (LASSO) algorithm to gradually select the optimal features. The feature extraction and analysis process are illustrated in Figure 2.

### 2.5 Pathologic assessment

Whole tumor specimens were placed in formalin solution and sent to a pathology laboratory. After staining with hematoxylin and eosin, histopathologic evaluation was performed by a pathologist with more than 10 years of experience according to the ISUP grading system to obtain tumor grading information.

### 2.6 Statistical analysis

The categorical variables were compared using the Chi-squared test, and the continuous variables were compared using the Mann–Whitney *U* test.

Classification was performed using the SVM model. Receiver operating characteristic (ROC) curve analysis was used to evaluate the prediction performance of the radiomic signature. The optimal cutoff value was selected as the point when the sensitivity plus specificity was maximal. The area under the curve (AUC) was calculated in both the training and validation sets. A *Z* test was then used to investigate the difference in the AUC of the 3 sets of results.

Intraclass correlation coefficients (ICCs) with 95% confidence intervals (95% CIs) were used to assess the continuous variable. ICC > 0.75 signifies good inter-rater agreement. The ICC calculation, Mann–Whitney *U* test, and Chi-squared test were performed using IBM SPSS Statistics (version 22.0; SPSS, Chicago, IL). The confidence level was maintained at 95% and *P*-values of <.05 were considered significant. The *Z* test was performed using MedCalc (version 15.6.1). When *Z* > 1.96 and *P* < .05, the difference between the 2 groups was considered to be statistically significant. Dimensionality analysis, classifier construction, and ROC analysis were performed at Radcloud (Huiying Medical Technology Co, Ltd). Computer-generated random numbers were used to assign 80% of the ROIs as the training data and the other 20% as the validation data.

## 3 Results

The study included 155 patients in the low-grade group (51 at grade I, 104 at grade II) and 72 in the high-grade group (59 at grade III, 13 at grade IV). The gender and age characteristics in Table 1 show that there was no significant correlation between patient characteristics and grade.

**Table 1:**
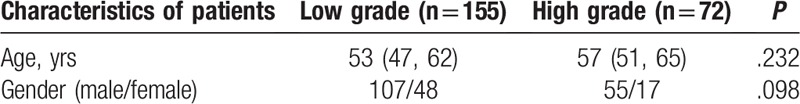
Characteristics of patients.

**Figure 3:**
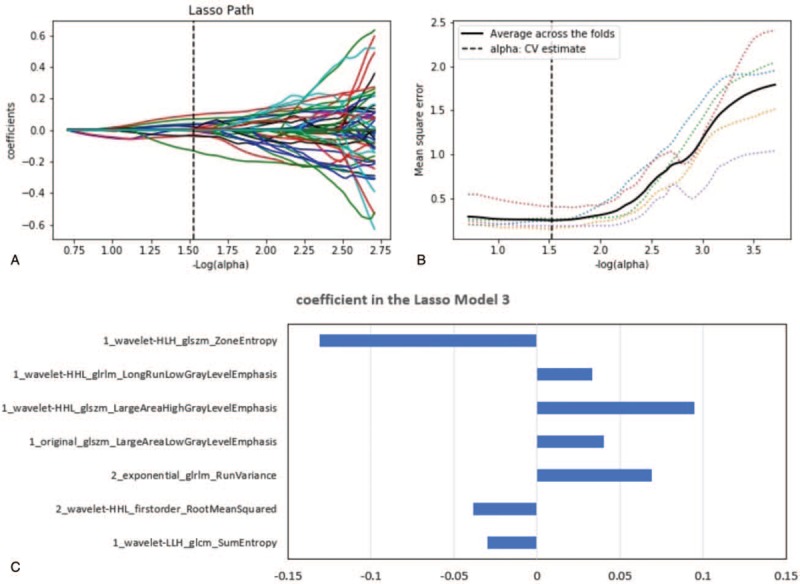
LASSO algorithm for feature selection in model 3. (A) LASSO path. (B) MSE path. (C) Coefficients in LASSO model. Using LASSO model, 7 features corresponding to the optimal alpha value were selected. 1 and 2 represent the corticomedullary phase and nephrographic phase, respectively.

We obtained 609, 590, and 1199 features using the variance threshold for the corticomedullary, nephrographic, and 2-phase joint samples, respectively, and then obtained 138, 51, and 189 features using the select K best method. Finally, 7, 5, and 7 optimal features were screened using the LASSO algorithm. In the 2-phase joint sample, the 7 features selected were the Zone Entropy, Long Run Low Gray Level Emphasis, Large Area High Gray Level Emphasis, Sum Entropy, Large Area Low Gray Level Emphasis, Root Mean Squared, and Run Variance. The LASSO path, mean squared error (MSE) path, and characteristic coefficients in the LASSO model are shown in Figure 3. The inter-rater agreement ranged from 0.973 to 0.998 for the above image features.

**Figure 4:**
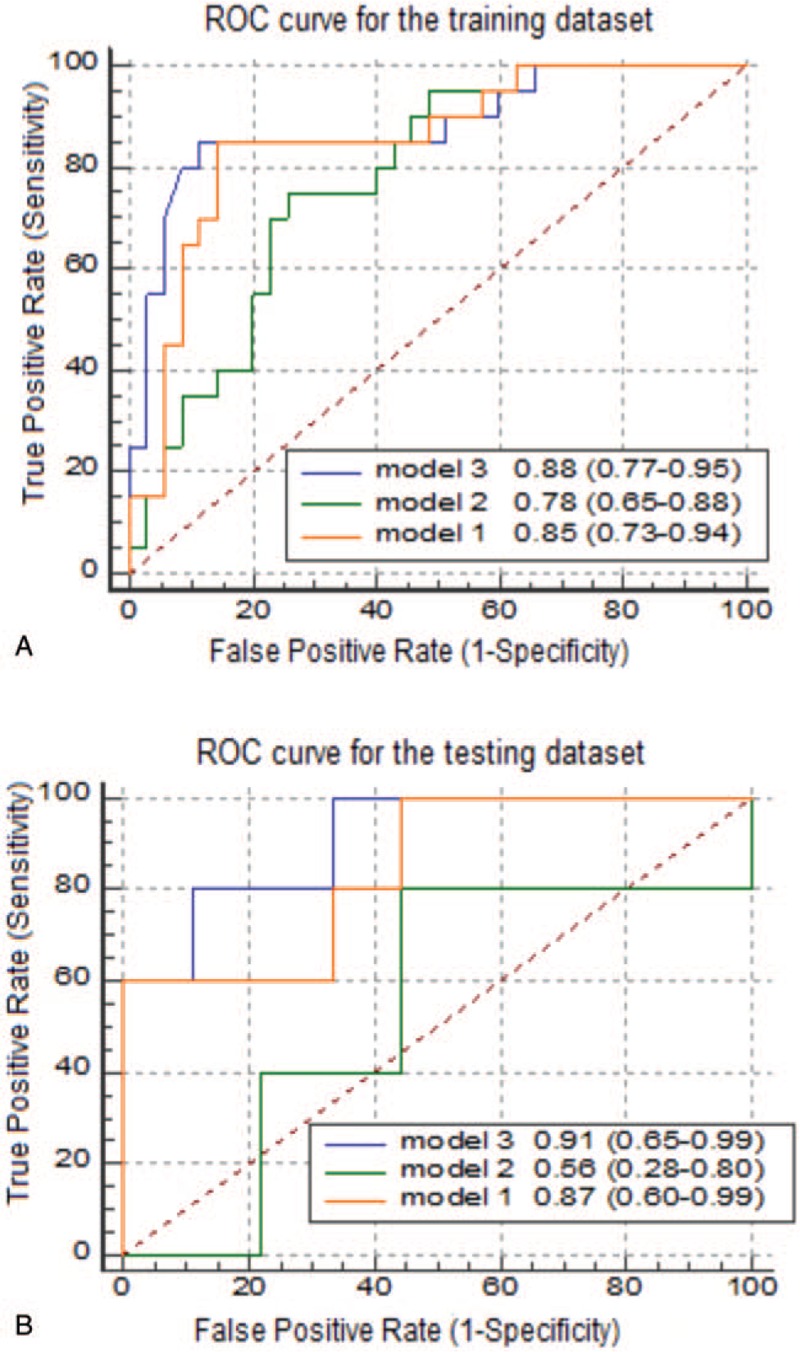
Different receiver operating characteristic (ROC) curves for support vector machine classifiers. Comparison of ROC curves between model 1, model 2, and model 3 for the prediction of ISUP grading in the (A) training and (B) testing data sets.

The model was constructed using the selected features of the corticomedullary, nephrographic, and 2-phase joint samples, named Model 1, Model 2, and Model 3, respectively. Model 3 achieved the best training results with the SVM classifier. The AUCs of the training and validation sets were 0.88 (95% CI: 0.77–0.95; sensitivity 0.85, specificity 0.89) and 0.91 (95% CI: 0.65–0.99; sensitivity 0.83, specificity 0.89), respectively. The ROC curves of each group are shown in Figure 4.

The results of the classifiers constructed in models 1 and 2 show that the prediction accuracy of the nephrographic-phase data was poor, with the AUC of the validation set reaching only 0.56. For the classifier constructed using corticomedullary phase data, although the AUC of the validation set reached 0.87, its specificity was low at only 0.67. The AUC was bigger in model 3 than in model 2 (*Z* = 2.277, *P* = .023). This indicates that the difference between the model constructed using the nephrographic phase data and the result of the 2-phase joint model is statistically significant. No significant AUC difference was found between model 1 and model 3 (*P* > .05). The ROC curve analysis results for the training and verification sets are presented in Table 2.

**Table 2:**
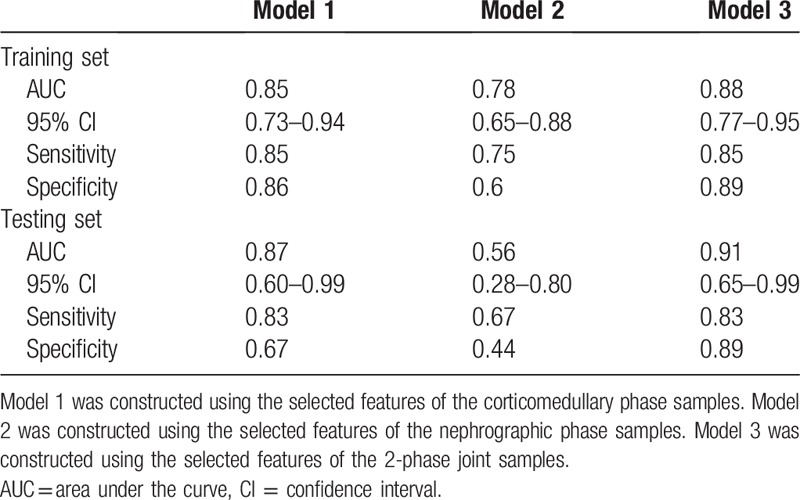
Receiver operating characteristic analysis by support vector machine classifiers of training and testing sets.

## 4 Discussion

In this study, we sought to investigate whether CT-based radiomic models could distinguish between high- and low-grade ccRCC in the context of the updated nuclear grading standards. We found that the SVM model established using radiomic features provides an effective identification of the simplified ccRCC grading.

Previous studies have shown that CT-based radiomics is a valuable tool in distinguishing high- and low-grade ccRCC. The radiomics model with texture features constructed by Ding et al exhibits high prediction accuracy in identifying the grading of ccRCC, with an AUC value of 0.771.^[6]^ Their results were superior to those obtained using CT image features or the RENAL nephrometry score for high- and low-grade ccRCC predictions, which achieved AUC values of 0.70 and 0.73, respectively.^[5,7]^ In the present study, the AUC value of the model based on 2-phase joint features reached 0.91, which is somewhat higher than the AUC results of previous studies. This may be due to 2 reasons. Firstly, the evaluation criteria for the 2 groups of grading systems are different.^[12]^ The details of the ISUP and Fuhrman grading system are shown in Table 3. The ISUP grading system in which nuclear size is taken as the classification standard, enabling each grade to be effectively distinguished, was used in this study. All of the above studies used the Fuhrman grading criteria, which consider nuclear size, nuclear shape, and nucleolar prominence as references.^[9]^ Subjective ratings can only be made by pathologists when the grading of the 3 parameters is divergent.^[5]^ This directly leads to inaccurate grading and poor reproducibility of grading results.^[4,10,11,18]^ Secondly, unlike the work of Ding et al, this study did not include nontexture features, and there were significant differences in the choice of dimensionality reduction analysis methods. Another study showed that the high and low grades of ccRCC could be predicted using a variety of models, of which the SVM model achieved the highest accuracy (AUC value of 0.860).^[19]^ However, this study was based on the Fuhrman grading system. This may be the main reason for the difference from the results of the present study. These 3 studies have reached a similar conclusion that CT texture analysis is a useful and promising method for noninvasive prediction of ccRCC pathologic nuclear grading.

**Table 3:**
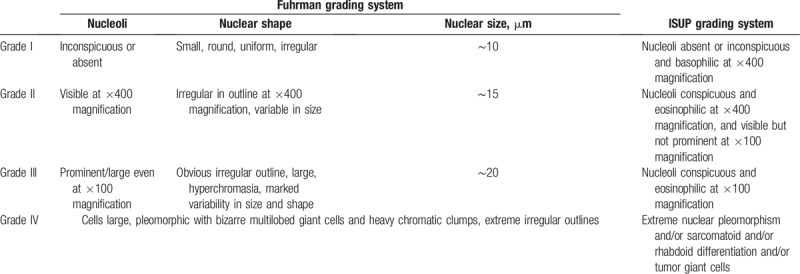
International Society of Urologic Pathology (ISUP) and Fuhrman grading system.

The results of the present study show that the 3 models constructed using radiomic features can achieve higher accuracy in terms of predicting the ISUP grades. There was no statistically significant difference between model 3, constructed using the 2-stage joint data, and model 1, constructed using the corticomedullary phase data. This demonstrates that the value of the nephrographic phase data is limited in the prediction of the ISUP grading. Moreover, model 3 obtained the highest AUC value of 0.91, and strong sensitivity and specificity values of 0.83 and 0.89, respectively. Therefore, in clinical practice, we recommend that image data should be used as comprehensively as possible to predict the ISUP grading of ccRCC.

This study had the following limitations. First, the sample size in this experiment was relatively small. Although a positive result has been obtained, a larger sample of experiments is still needed for verification in the future. Second, we only used the ROI of the largest cross-section to extract features, and did not perform data analysis of the whole tumor ROI. However, previous studies have shown that the radiomic features of 2-dimensional data extraction result in better performance in the prognosis of NSCLC.^[20]^ Moreover, we performed whole-tumor delineation in a preliminary experiment, but it was difficult to obtain high prediction accuracy in the test cohort in the 2-phase joint model, and the specificity of each group model was low.

Overall, our research may have important clinical implications because, for patients with ccRCC, different grades represent different postoperative cancer-free survival rates and different risks of metastasis.^[11]^ According to our present study, models constructed using radiomic features have comparable performance to percutaneous biopsy in predicting ISUP grading. Thus, accurate nuclear grading prediction information can be obtained with noninvasive CT examination, and the risk caused by needle biopsy can be avoided.^[21]^ Moreover, the method allows for repeated noninvasive assessment of nuclear grading during follow-up. With the development of immunotherapy, this provides a new direction for the treatment of RCC. However, different immune characteristics will also have an impact on the adoption of immunotherapy. This is confirmed in the study of immunotherapy for RCC by Gigante et al and Cavalcanti et al.^[22,23]^ However, it is still unknown whether radiomics can help in the identification of immune features. This is worth exploring, and will be our next research direction.

## 5 Conclusion

The results from this study show that, in the context of the updated ccRCC pathologic nuclear grading system, an SVM model based on CT images can accurately distinguish between the high and low grades of the ISUP system.

## Author contributions

**Conceptualization:** Xiaoqing Sun, Yuqing Jiang.

**Data curation:** Xiaoqing Sun, Ziqi Huo, Heng Liu, Tongxu Shen, Feng Pan, Yuqing Jiang.

**Formal analysis:** Wenhui Li.

**Investigation:** Mengchao Zhang.

**Methodology:** Xiaoqing Sun.

**Resources:** Kai Xu.

**Supervision:** Xiaoqing Sun, Kai Xu, Mengchao Zhang.

**Visualization:** Lin Liu.

**Writing – Original Draft:** Xiaoqing Sun.

**Writing – Review & Editing:** Mengchao Zhang.
